# Moral

**Published:** 1916-11-18

**Authors:** 


### MORAL.

### Its First Reinforcement.

The pair of words, 'mor′al' and 'moral′' is an example, not very infrequent in our language, of two words that are spelt in the same way, but differ widely in meaning according as the stress is laid on the first syllable or the last. Other examples are 'rec′ord' and 'record′,' 'Aug′ust' and 'august′,' 'in′sult' and 'insult′,' 'per′fect' and 'perfect′.' For the last two or three years we have been hearing a great deal about 'moral′,' and to many the word is new, and many have but a hazy notion of its meaning, though everyone has by this time a sense of its tremendous importance. Certainly the correspondents with our armies and the expert commentators at home are not all acquainted with the meaning of 'moral,' and some of them misconceive its meaning as much as they misconceive the meanings of 'decimate' and 'annihilate,' which they use as interchangeable and synonymous words, both meaning 'seriously damage.' It need scarcely be pointed out to readers of The Hospital that to decimate means to kill every tenth man, and to annihilate means to reduce to nothing, so that if a regiment is decimated it has lost one-tenth of its strength and nine-tenths remain, whereas if it is annihilated nothing at all remains. Everyone with any pretence to be considered educated knows this, but even some who do know this have no very definite idea of what is meant by the moral of an army or of a nation.

### The Basis of a High Moral.

Of course, 'moral′' has nothing to do with morality or rectitude of conduct. Even the special correspondents know this; but some of them write as if the moral of soldiers or citizens meant their courage, or their elation of spirits, or their willingness to sacrifice their lives, or their cheerfulness under discomfort, or their fortitude under suffering, or their dash and intrepidity of attack, or their stubbornness in defence. No doubt moral includes these qualities, but none of them is quite the equivalent of moral. Moral may be possessed and displayed as much on the march as on the field of battle, as much by non-combatants as by combatant troops, as much by the civil population as by the army itself. It would be nearer the mark to say that moral means confidence in ultimate victory, which would contribute to high spirits, to bravery, to cheerfulness, fortitude, intrepidity, and stubbornness. Confidence in ultimate victory is no doubt a frequent accompaniment of high moral, and when it is felt, contributes more than almost anything else to high moral; but it is not quite the same thing as high moral, for there have been many instances in history of heroic refusals to surrender, even when victory was hopeless, and when the alternative to surrender was extermination. It would be impossible to deny to such a defence the inspiration of a high moral. No. What is meant by a high moral is *a stubborn determination to endure to the end.* That is the basis of a high moral. If there is a confidence in victory, so much the better. In that case stubborn endurance is the less needed; but it must be there. It lies at the foundation of moral, and without it the confidence in victory is a bruised reed. In victory moral is displayed, no doubt, but in victory it is not needed. Moral is tested not in victory, but in defeat. A victorious army has a good moral of course, but it has little need of moral, for it has comparatively little to endure, and the elation of victory supplies the place of moral. It is when victory is in suspense, is doubtful, or is definitely lost that moral is needed, and that an army or a nation has an opportunity of displaying its moral. Even a victorious nation is wanting in moral if, from weariness of war, it concludes an indecisive peace. It has shown itself wanting in stubborn endurance.

### The Encouragement of Moral.

Confidence in ultimate victory, though it is not itself high moral, yet contributes greatly to high moral; for it encourages stubborn endurance by holding out an ultimate reward. Moreover, as it is easier to endure little than to endure much, and as confidence in victory holds out the hope that there will be comparatively little to endure, it is the object of every commander to give to his troops confidence in ultimate victory. It is common knowledge, indeed it is commonplace, that nothing contributes so much to victory as high moral. Troops whose moral is good will achieve impossibilities—troops whose moral is bad are at the mercy of the enemy. Superiority of numbers, better discipline, excellence of position, abundance of munitions of war, skilful leadership, have all been proved again and again to be worthless in the absence of a good moral; and over and over again a good moral has won the victory in spite of disparity, sometimes of enormous disparity, in some or all of these respects. Hence every commander tries to give to his troops confidence in victory. Such confidence is acquired from one or more of the following sources, and the measures of the commander are taken accordingly.

The first reinforcement of moral comes from a belief in the righteousness of the cause that is fought for. In modern times, when the rank and file are disinclined to surrender their consciences into the hands of their leaders as they freely did even in Elizabethan times, every man is uneasy in his conscience and suffers in his moral if he has doubts of the righteousness of the cause for which he is fighting. Every Government and every Headquarters Staff knows this, and therefore the aim of every Government is to represent the war in which it has engaged, or is about to engage, as righteous and just. The simplest way to do this is to represent the war as an unavoidable war, a defensive war undertaken in opposition to unprovoked and unrighteous aggression, and this is the method employed by the Germans in the present war. The German is by nature a slave, and does what he is bidden without regard either to its sense or to its morality; but even the German likes to have a good cause, and therefore at the outset of both the war of 1870 and the present war the German Government was at pains, by enormous lying and garbling or suppression of documents, to make its army believe that its cause was righteous. One would not suppose that German soldiers would care a straw whether their cause was righteous or not as long as it was ordered by their masters; nor would they as long as they were victorious; but it is in defeat, whether local or general, that moral is most needed and is most apt to suffer; and no army is secure against local defeats. Hence the sedulous care of the rulers of Germany to impress upon their soldiers and the nation that Germany had been attacked wantonly and without provocation.

*(To be continued.)*

